# Pathway Network Analyses for Autism Reveal Multisystem Involvement, Major Overlaps with Other Diseases and Convergence upon MAPK and Calcium Signaling

**DOI:** 10.1371/journal.pone.0153329

**Published:** 2016-04-07

**Authors:** Ya Wen, Mohamad J. Alshikho, Martha R. Herbert

**Affiliations:** 1 TRANSCEND Research, Neurology Department, Massachusetts General Hospital, Charlestown, Massachusetts, United States of America; 2 Harvard Medical School, Harvard University, Boston, Massachusetts, United States of America; 3 Higher Synthesis Foundation, Cambridge, Massachusetts, United States of America; The George Washington University, UNITED STATES

## Abstract

We used established databases in standard ways to systematically characterize gene ontologies, pathways and functional linkages in the large set of genes now associated with autism spectrum disorders (ASDs). These conditions are particularly challenging—they lack clear pathognomonic biological markers, they involve great heterogeneity across multiple levels (genes, systemic biological and brain characteristics, and nuances of behavioral manifestations)—and yet everyone with this diagnosis meets the same defining behavioral criteria. Using the human gene list from Simons Foundation Autism Research Initiative (SFARI) we performed gene set enrichment analysis with the Kyoto Encyclopedia of Genes and Genomes (KEGG) Pathway Database, and then derived a pathway network from pathway-pathway functional interactions again in reference to KEGG. Through identifying the GO (Gene Ontology) groups in which SFARI genes were enriched, mapping the coherence between pathways and GO groups, and ranking the relative strengths of representation of pathway network components, we 1) identified 10 disease-associated and 30 function-associated pathways 2) revealed calcium signaling pathway and neuroactive ligand-receptor interaction as the most enriched, statistically significant pathways from the enrichment analysis, 3) showed calcium signaling pathways and MAPK signaling pathway to be interactive hubs with other pathways and also to be involved with pervasively present biological processes, 4) found convergent indications that the process “calcium-PRC (protein kinase C)-Ras-Raf-MAPK/ERK” is likely a major contributor to ASD pathophysiology, and 5) noted that perturbations associated with KEGG’s category of environmental information processing were common. These findings support the idea that ASD-associated genes may contribute not only to core features of ASD themselves but also to vulnerability to other chronic and systemic problems potentially including cancer, metabolic conditions and heart diseases. ASDs may thus arise, or emerge, from underlying vulnerabilities related to pleiotropic genes associated with pervasively important molecular mechanisms, vulnerability to environmental input and multiple systemic co-morbidities.

## Introduction

While there remains controversy regarding the relative contributions of artifact and true increase to the rising prevalence of ASDs, there is no question that these spectrum conditions have major human, public health and economic impacts. They also pose major challenges to research, since while ASDs are defined by social impairments, communication difficulties, and restricted, repetitive, and stereotyped patterns of behavior, there are no pathognomonic biological features universally present in people meeting the behavior-level diagnostic criteria, and many other biological features have been identified in subgroups both in the brain and systemically whose contributions to the development and ongoing characteristics of ASDs are not clearly understood.

As of December 2014, the SFARI Gene-Human Gene Module recorded 667 human genes implicated as relevant to ASDs. These genes are widely expressed and are involved in a variety of biological activities. To make full biological sense of this large number and diversity of genes, it is important both to consider the full range of their biological activities, and to identify processes with higher likelihood of substantial relevance to ASDs.

In this study we chose to consider the accumulated set of genes as a whole, and to utilize established databases and analytical processes to seek patterns and connections within this large aggregate. To do this we expanded the framework from just genes to also include the contexts, reactions and processes in which they participate. We used enrichment analysis to identify 1) pathways in which ASD-associated genes were over-represented, 2) biological features for which ASD-associated genes were particularly present, and 3) overlaps and convergences amongst the several types of analyses that we performed. Future studies may add more nuances to the analyses we report, including addressing biases that may be present at various stages of the process ranging at least from gene discovery to database construction. For the present study we did not introduce any biases from favoring certain types of biological processes or domains over others, and we did not utilize any weighting systems, as we could not locate any established or fully developed weighting methods that could do justice to all the different varieties of ASDs, considering the marked heterogeneity this diagnosis encompasses. We aimed simply to let the data speak through the patterns and relationships that emerged from performing standard analyses, regardless of whether the resultant findings were consistent with areas of predominant focus in the existing literature. To our knowledge the aggregate of genes associated with ASD, along with their functions, contexts and pathway networks, have not previously been analyzed in this manner.

The networks in which cellular processes often function, which involve sets of gene products acting in concert, can be mapped as pathways. Since there are many overlaps among pathways, identifying convergent molecular pathways in which multiple candidate genes are involved has been discussed for years [1], as this may be an effective way to gain insight into the underlying molecular basis of ASDs. There are a number of studies that aim to discern potential important pathways to ASDs, whether by developing protein interaction networks [2,3] or gene co-expression networks [4], or by identifying specific affected pathways from ASD genetic databases [5]. However, there are very few studies that have systematically investigated pathways or pathway networks that may link to ASDs, and to our knowledge there are no prior studies looking at pathway-pathway interactions at the functional level in ASDs.

In this study we developed an ASD pathway network and performed a systematic analysis of the network. With this approach we aimed to expand our understanding to include not only genes but also the function of gene products, as well as the networking of reactions and interactions. In thus generating a coordinated presentation whose depictions take account of gene-pathway coherence, pathway-pathway interactions, and disease-disease relationships, our objective was to provide a framework and clues for further investigations.

## Materials and Methods

### Gene set enrichment analysis

To identify a variety of pathways and biological signatures, we investigated enrichment within the Human Gene Module of SFARI Gene. ASD genes were downloaded from SFARI Gene–Human Gene (https://gene.sfari.org/autdb/HG_Home.do, S1 Table), and investigated in the freely available Molecular Signatures Database, MSigDB [6] v4.0 (http://www.broadinstitute.org/gsea/msigdb/index.jsp). We applied the “Compute Overlaps” tool from MSigDB website, under “Investigate gene sets” category, which uses the hypergeometric distribution to examine how SFARI genes and other gene sets may overlap. Gene symbol was used as gene identifier to import the gene set. MSigDB is a collection of annotated gene sets for use with Gene Set Enrichment Analysis (GSEA) software [6]. Here we used the MSigDB collections derived from the KEGG Pathway Database (http://www.genome.jp/kegg/pathway.html) and the GO Consortium (http://geneontology.org/). The GO dataset provides a central collection of the attributes already known and assigned to specific genes.

### Overlaps and ranking

The overlaps between SFARI genes and KEGG gene sets (derived from the KEGG pathway database and each of the sub-collections of GO gene sets, (i.e., the Biological Processes (BP), cellular component (CC) and molecular function (MF) ontologies), were computed individually. Data obtained from this procedure can be found in S2 Table; the top 50 enriched gene sets with false discovery rate (FDR) q-value below 0.05 were listed, ranked by p-values (low to high). GSEA allows us to choose the enriched groups from top 10, 20, 50, and 100. We made the judgment to choose 50 because it is a moderate number for pathway network analysis–large enough to build an interaction network and small enough to avoid overly diluting the ASD specificity of the genes. Further information regarding the “Compute Overlaps” tool can be found on the “Help” page under Molecular Signatures Database, under “Help with Investigating Gene Sets” here: http://software.broadinstitute.org/gsea/msigdb/help_annotations.jsp—overlap (one needs to register and login to use this link).

### Redundancy control for the enriched pathways

Highly overlapped pathways may cause redundancy, and this might bias the results of analysis. To minimize the impact of such redundancy among our gene sets, Redundancy Control in Pathway Databases (ReCiPa) [7] was applied to the top 50 pathways resulting from the enrichment analysis. The ReCiPa package was downloaded from the url described in the original paper (http://cran.r-project.org/web/packages/ReCiPa/) and installed and loaded in R (http://www.r-project.org/). The KEGG pathway database file was downloaded from MSigDB Downloads (http://www.broadinstitute.org/gsea/downloads.jsp). We used the enriched 50 KEGG pathways as our input database. Highly overlapped pathways were merged into collections (setting Max = 0.85, Min = 0.10). For each merged collection, the p-value from the dominant pathway (pathways with lowest p-values) was used as the p-value for the particular collection.

### Pathway-pathway and pathway-gene interactions

In cells, pathways interact with each other rather like street-meets-street in a city map. In order to obtain a systems overview of how these pathways function together, it is of great value to generate a pathway network, which can be done by taking account of the connections/interactions between pathways. One pathway may interact with another for multiple reasons: shared genes or compounds, interactions or reactions between genes or compounds, signal transductions, etc. We chose to work with KEGG Pathway Database, which documents these many types of influences and interactions, to help us generate this pathway network overview, because it is a comprehensive and frequently updated database that represents our present knowledge of pathways and the known interactions amongst pathways based on accumulated research findings. Pathway-pathway interactions are represented in KEGG in each pathway map by displaying the name of an interacting pathway in that map. For example, if one pathway appears at the certain location in the map of another pathway, this suggests that these two pathways were interacting in relation to the activities or functions represented in that part of the map, or that one was involved in a certain reaction or process of another. On this basis, we determined the pathway-pathway interactions by tabulating these instances. For the merged collections, if one pathway not in the collection interacted with at least one of the pathways in the collection, this pathway was considered to be interacting with the collection. For the pathway “pathways in cancer” (http://www.genome.jp/kegg-bin/show_pathway?map05200, or go to KEGG, search for “pathways in cancer”, and find the map05200), other cancer pathways (such as “glioma”, “thyroid cancer” etc.) which were listed on the bottom of the KEGG pathway map, did not count as interacting with the “pathways in cancer”, as they are not actually interacting with each other but only being listed in the map.

For pathway-pathway interactions, the total number of interactions was tabulated for each pathway/collection (S3 Table), and then visualized in a network diagram.

To investigate pathway-gene interactions characterizing the involvement of each ASD gene in the pathway network, the number of pathways in which each gene participates was counted, and the total number of pathways was tabulated for each gene.

### Pathway network analyses

We then examined the pathway network for patterns of more significant or common interactions and functional impacts, aiming to highlight ways that these pathways and processes might particularly contribute to ASD pathology at the function level. We examined the pathway network to identify any “hub” (most interactive) pathways, most involved genes, possible links between the pathway network and GO groups. Then we looked for any convergences between the predominant pathways, genes and GO groups thereby identified. Out of this examination we identified and depicted convergent mechanisms.

## Results

### Most enriched pathways

659 out of 667 ASD genes from SFARI Genes were identified in MSigDB v4.0. We did not include those genes that were not identified in MSigDB in our analysis. The top 50 enriched pathways (from the 659 genes) were chosen from the computation of overlaps with KEGG gene sets (S2 Table). These top 50 pathways were sorted by p-values regarding significance of overlaps; the “calcium signaling pathway” (p-value 2.84E-29) and “neuroactive ligand-receptor interaction” (p-value 2.87E-29) stand out with their markedly stronger statistical significance (S2 Table).

### Enriched pathways groupings and redundancy reduction

The KEGG Pathway Database contains diversely named pathways that overlap with one another to varying degrees. In our data, among our 50 pathways, we identified 13 enriched cancer pathways that might overlap amongst themselves at multiple levels. The application of ReCiPa [7] merged highly overlapped pathways and generated two collections. By merging 2 cardiac disease pathways a single *collection cardiac disease* was generated, and by merging 10 cancer pathways a single *collection cancer* was generated. The final yield from 50 pathways was a list of 40 pathways/collections, which we used for further analysis (Table 1).

**Table 1:** Pathway groupings and pathway-pathway interactions.

Description	Interactions	ASD genes	P-values	# (%) Pathways
**Functional pathways**				30 (75%)
Cell signaling				9 (22.5%)
**Calcium signaling pathway**	12	35	2.84E-29	
**MAPK signaling pathway**	20	22	6.59E-11	
Cell adhesion molecules (CAMs)	1	16	1.14E-10	
Wnt signaling pathway	9	15	5.70E-09	
mTOR signaling pathway	4	10	3.16E-09	
Vascular smooth muscle contraction	1	14	1.28E-09	
ErbB signaling pathway	5	11	5.07E-08	
ECM-receptor interaction	4	10	3.66E-07	
GnRH signaling pathway	3	10	2.05E-06	
Cell structure / Transport				5 (12.5%)
Focal adhesion	5	21	1.98E-12	
Gap junction	4	14	4.50E-11	
Regulation of actin cytoskeleton	8	18	2.97E-09	
Adherens junction	5	11	1.04E-08	
Endocytosis	0	15	7.61E-08	
Metabolism				8 (20%)
Melanogenesis	4	16	1.62E-12	
Lysine degradation	1	8	1.93E-07	
Ubiquitin mediated proteolysis	6	13	1.11E-07	
Tyrosine metabolism	2	7	2.11E-06	
Phenylalanine metabolism	1	5	4.38E-06	
Aldosterone-regulated sodium reabsorption	1	7	2.11E-06	
Tryptophan metabolism	0	7	1.49E-06	
Arginine and proline metabolism	1	7	1.19E-05	
Neural				5 (12.5%)
**Neuroactive ligand-receptor interaction**	2	41	2.87E-29	
Long-term potentiation	3	17	1.46E-16	
Neurotrophin signaling pathway	4	17	4.06E-12	
Long-term depression	1	11	4.88E-09	
Axon guidance	2	13	4.96E-08	
Immune				3 (7.5%)
Leukocyte transendothelial migration	3	10	8.35E-06	
Chemokine signaling pathway	4	12	2.19E-05	
B cell receptor signaling pathway	4	8	1.24E-05	
**Disease Pathways**				10 (25%)
Cancer				4 (10%)
*Collection cancer*: *Acute myeloid leukemia*, *Bladder cancer*, *Chronic myeloid leukemia*, *Colorectal cancer*, *Endometrial cancer*, *Melanoma*, *Non-small cell lung cancer*, *Pathways in cancer*, *Prostate cancer*, *Small cell lung cancer*	9	37	2.95E-18	
Renal cell carcinoma	2	11	4.88E-09	
Thyroid cancer	3	7	1.43E-07	
Glioma	4	8	4.21E-06	
Cardiac disease				2 (5%)
Arrhythmogenic right ventricular cardiomyopathy (ARVC)	3	12	9.21E-10	
*Collection cardiac disease*: *Dilated cardiomyopathy*, *Hypertrophic cardiomyopathy (HCM)*	1	13	7.64E-10	
Metabolic disease				1 (2.5%)
Type II diabetes mellitus	0	8	3.29E-07	
Neurodegenerative disease				3 (7.5%)
Alzheimer's disease	2	13	1.15E-06	
Amyotrophic lateral sclerosis (ALS)	1	7	1.05E-05	
Huntington's disease	1	12	1.68E-05	

Interactions = number of other pathways in the network with which each pathway interacted. ASD genes = number of ASD genes in each pathway. #(%) Pathways = number of pathways in each group and that group’s percentage of the total network. Most significant pathways are indicated in bold. Collections are indicated in italic.

### Subgrouping of pathways/collections

We subdivided the 40 pathways/collections into 30 function-related pathways and 10 disease-related pathways, and further sub-grouped them according to their characteristics (Table 1):

1. The 30 function-related pathways were sub-grouped into 5 sub-categories: cell signaling (encompassing 9 pathways under 3 groups: signal transduction, signaling molecules and interaction), cell structure/transport (encompassing 5 pathways under 3 groups: cell motility, communication and cellular transport), metabolism (encompassing 8 pathways under 3 groups: amino acid metabolism, protein degradation, and synthesis), neural (encompassing 5 pathways with no subgroups), and immune (encompassing 3 pathways with no subgroups).
2. The ten disease-related pathways/collections were sub-grouped into 4 sub-categories: cancer (4 of 10), cardiac disease (2 of 10), metabolic diseases (1 of 10) and neurodegenerative diseases (3 of 10).

**Fig 1:**
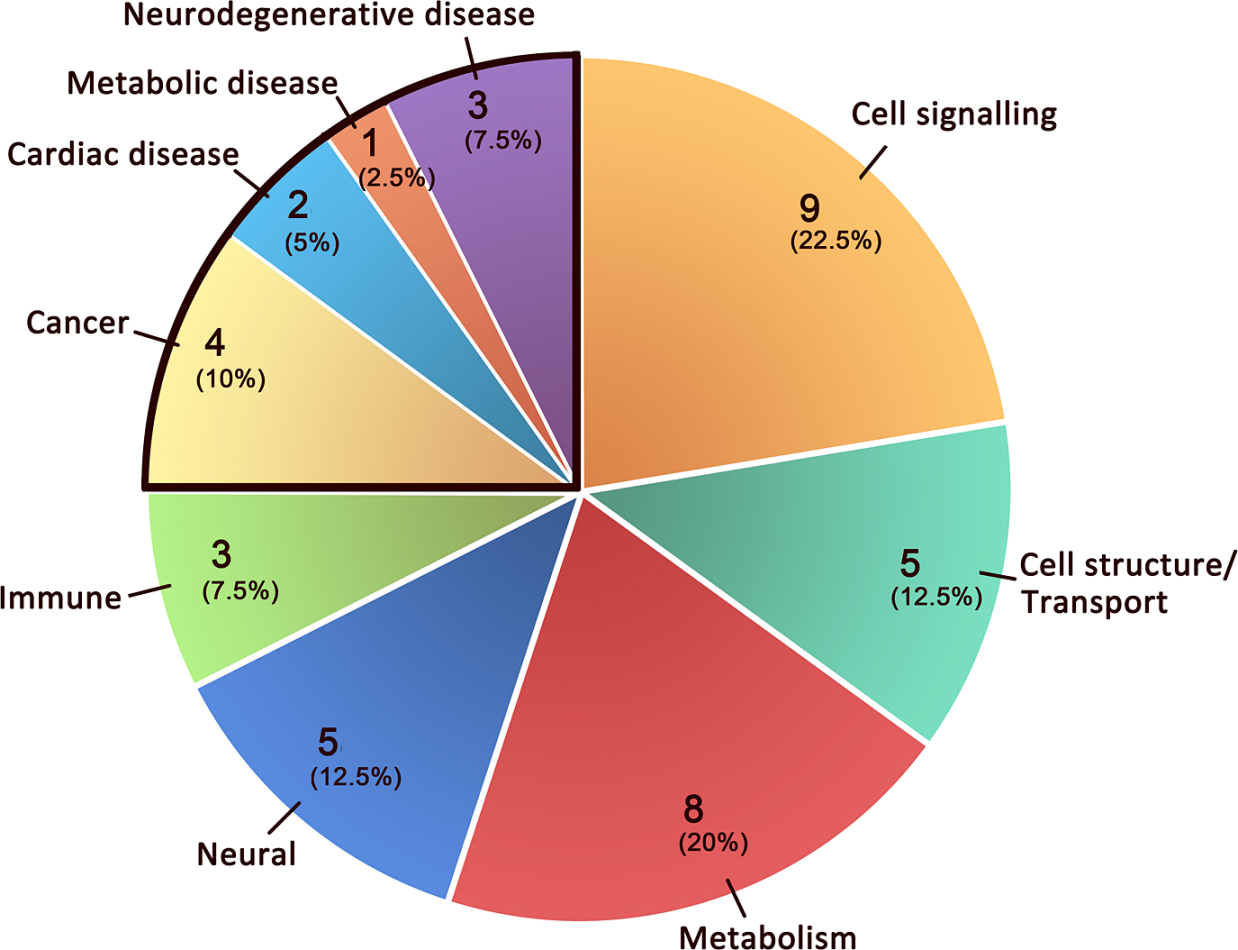
Pathway groupings. Pathways were grouped into subsets of two classes. Pathways in the Disease class are surrounded by a black border; the remainder of the pie chart sections are in the Functional class. Labels indicate pathway groupings (number of pathways in grouping/percentage of total number of pathways).

Fig 1 displays the proportional contribution of pathway groups and subgroups to the overall ASD pathway network, and shows that the largest proportions of pathways were contributed by the cell signaling subgroup in the function-related pathways group and the collection cancer in the disease-related pathways group.

### Gene Ontology grouping and overlaps between most enriched pathways

The results of ASD gene enrichment analysis in MSigDB with GO gene sets were divided into three GO groups: biological processes (BP) (most enriched for “nervous system development”, p-value 2.08E-61), cellular components (CC) (most enriched for “plasma membrane” p-value 1.19E-60), and molecular functions (MF) (most enriched for “receptor activity”, p-value 3.07E-28) (Table 2).

**Table 2:** Enrichment analyses for gene ontologies.

	Genes in gene set	ASD genes	P-value	FDR q-value
**Biological Processes Gene Set Names**				
NERVOUS_SYSTEM_DEVELOPMENT	385	75	2.08E-61	1.72E-58
MULTICELLULAR_ORGANISMAL_DEVELOPMENT	1049	100	1.58E-51	6.50E-49
SYSTEM_DEVELOPMENT	861	91	1.29E-50	3.55E-48
ANATOMICAL_STRUCTURE_DEVELOPMENT	1013	96	3.30E-49	6.80E-47
SIGNAL_TRANSDUCTION	1634	106	4.95E-39	8.17E-37
SYSTEM_PROCESS	563	57	7.73E-31	1.06E-28
CENTRAL_NERVOUS_SYSTEM_DEVELOPMENT	123	30	2.94E-28	3.46E-26
BIOPOLYMER_METABOLIC_PROCESS	1684	92	3.38E-28	3.48E-26
NEUROLOGICAL_SYSTEM_PROCESS	379	45	1.78E-27	1.63E-25
CELL_DEVELOPMENT	577	52	8.13E-26	6.71E-24
TRANSMISSION_OF_NERVE_IMPULSE	189	32	9.78E-25	7.33E-23
SYNAPTIC_TRANSMISSION	174	30	1.64E-23	1.13E-21
BIOPOLYMER_MODIFICATION	650	50	8.04E-22	5.10E-20
CELL_CELL_SIGNALING	404	40	1.33E-21	7.68E-20
PROTEIN_MODIFICATION_PROCESS	631	49	1.40E-21	7.68E-20
**Cellular Components Gene Set Names**				
PLASMA_MEMBRANE	1426	125	1.19E-60	2.77E-58
PLASMA_MEMBRANE_PART	1158	112	1.62E-58	1.88E-56
MEMBRANE	1994	142	7.02E-58	5.45E-56
MEMBRANE_PART	1670	127	1.23E-54	7.15E-53
INTRINSIC_TO_PLASMA_MEMBRANE	991	91	1.78E-45	8.31E-44
INTEGRAL_TO_PLASMA_MEMBRANE	977	90	4.39E-45	1.70E-43
INTRINSIC_TO_MEMBRANE	1348	102	2.20E-43	7.31E-42
INTEGRAL_TO_MEMBRANE	1330	100	2.81E-42	8.19E-41
CYTOPLASM	2131	112	6.37E-33	1.65E-31
CYTOPLASMIC_PART	1383	75	7.47E-23	1.74E-21
PROTEIN_COMPLEX	816	53	9.26E-20	1.96E-18
MACROMOLECULAR_COMPLEX	945	57	1.14E-19	2.21E-18
NUCLEUS	1430	71	1.47E-19	2.64E-18
CYTOSKELETON	367	31	4.18E-15	6.96E-14
INTRACELLULAR_ORGANELLE_PART	1192	56	1.31E-14	2.03E-13
**Molecular Functions Gene Set Names**				
RECEPTOR_ACTIVITY	583	55	3.07E-28	1.21E-25
TRANSMEMBRANE_RECEPTOR_ACTIVITY	418	43	8.59E-24	1.70E-21
G_PROTEIN_COUPLED_RECEPTOR_ACTIVITY	191	22	7.92E-14	9.15E-12
PHOSPHOTRANSFERASE_ACTIVITY_ALCOHOL_GROUP_AS_ACCEPTOR	334	28	1.08E-13	9.15E-12
PROTEIN_KINASE_ACTIVITY	285	26	1.16E-13	9.15E-12
KINASE_ACTIVITY	369	29	2.02E-13	1.34E-11
IDENTICAL_PROTEIN_BINDING	304	26	5.18E-13	2.93E-11
RHODOPSIN_LIKE_RECEPTOR_ACTIVITY	134	18	1.02E-12	5.07E-11
CATION_CHANNEL_ACTIVITY	119	17	1.57E-12	6.92E-11
GATED_CHANNEL_ACTIVITY	122	17	2.38E-12	9.42E-11
METAL_ION_TRANSMEMBRANE_TRANSPORTER_ACTIVITY	147	18	5.09E-12	1.83E-10
TRANSFERASE_ACTIVITY_TRANSFERRING_PHOSPHORUS_CONTAINING_GROUPS	424	29	6.34E-12	1.96E-10
ION_CHANNEL_ACTIVITY	149	18	6.42E-12	1.96E-10
TRANSMEMBRANE_TRANSPORTER_ACTIVITY	375	27	1.05E-11	2.97E-10
SUBSTRATE_SPECIFIC_CHANNEL_ACTIVITY	156	18	1.41E-11	3.71E-10
CYTOSKELETAL_PROTEIN_BINDING	159	18	1.94E-11	4.81E-10

Genes in gene set = total number of genes in the gene set. ASD genes = number of ASD genes in the gene set.

We noted that the three top enriched GO categories together contain one of the two above-mentioned most significant pathways, “neuroactive ligand-receptor interaction,” which is indeed involved in the “nervous system”, functions on “plasma membrane” and relates to “receptor activities”; that is, the most enriched pathway coincides with the most enriched GO groups in BP, CC and MF.

### Pathway-pathway interactions

We used the number of interactions a pathway has with other pathways in an overall network as an indicator of its level of involvement in the pathway network; this of course involves making a working assumption about the relevance of number of interactions to overall involvement; we proceeded in this way in order to generate hypotheses that will need to be tested by future investigations such as biological experiments and clinical studies. To display the results of the present analyses aimed at generating a provisional metric of extent of involvement of each pathway in the overall ASD pathway network we presented the counts of how many interactions each pathway in this network had with other pathways in the network, and evaluated the level of involvement of each pathway by the number of interactions it has (Table 1 and S3 Table).

**Fig 2:**
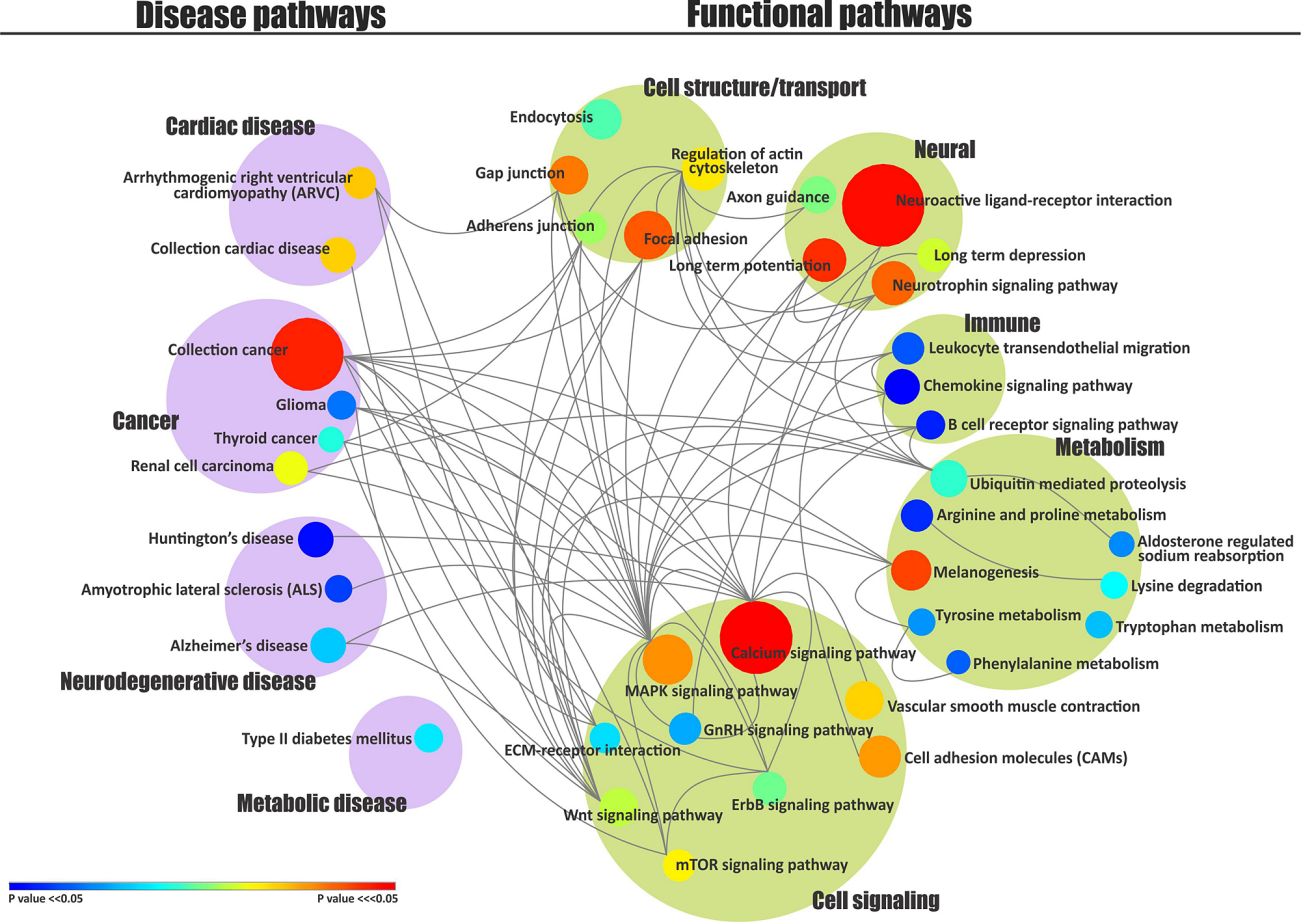
ASD pathway network. Pathways are grouped vertically under two classes, Disease and Function. The color of each node within the groupings represents the p-value of that pathway. The size of each node represents the number of ASD genes in that pathway. Interactions between pairs of pathways are indicated by edges. The pathway-pathway interaction matrix is in S3 Table.

Based upon this information regarding significance and connections between pathways contained in KEGG pathway maps, we generated a pathway-pathway interaction map by connecting all the pairs of interacting pathways and indicating significance by size and color of circles (Fig 2).

### ASD pathway network hub: MAPK signaling

The MAPK (mitogen-activated protein kinases, also known as ERK, extracellular signal-regulated kinases) signaling pathway interacts with half of the pathways in the network (20/40, Table 1), making this pathway the most inter-connected pathway in our map; within the terms of our analytical framework this identifies it as the main hub among the enriched pathways.

### Prominence of calcium signaling dysregulation

Calcium signaling pathway is not only the most enriched pathway but also very interactive in the ASD pathway network: it is the second most interactive pathway, interacting with 12 out of 40 pathways (Table 1). ASD genes in the calcium signaling pathway (*PRKCB*, *PLCB1*, *CACNA1C*, *CACNA1D*, and *CACNA1F*) are also highly involved in the pathway network, participating in 7–17 out of 40 pathways (Table 3). Additionally, Ca^2+^ (calcium ion) participates in 23 out of the 40 enriched pathways in reference to KEGG (http://www.genome.jp/dbget-bin/www_bget?C00076, or go to KEGG and search for C00076 –the compound number for calcium ion in KEGG).

**Table 3:** Involvement of ASD genes in the ASD pathway network.

Gene Symbol	Gene Description	# Pathways
MAPK1	mitogen-activated protein kinase 1	23
MAPK3	mitogen-activated protein kinase 3	23
HRAS	v-Ha-ras Harvey rat sarcoma viral oncogene homolog	18
PRKCB	protein kinase C, beta	17
BRAF	v-raf murine sarcoma viral oncogene homolog B1	14
PIK3CG	phosphoinositide-3-kinase, catalytic, gamma polypeptide	13
PIK3R2	phosphoinositide-3-kinase, regulatory subunit 2 (beta)	13
PLCB1	phospholipase C, beta 1 (phosphoinositide-specific)	11
GSK3B	glycogen synthase kinase 3 beta	10
CACNA1C	calcium channel, voltage-dependent, L type, alpha 1C subunit	9
CTNNB1	catenin (cadherin-associated protein), beta 1, 88kDa	8
CACNA1D	calcium channel, voltage-dependent, L type, alpha 1D subunit	8
CACNA1F	calcium channel, voltage-dependent, L type, alpha 1F subunit	7
CREBBP	CREB binding protein	7
EP300	E1A binding protein p300	7
ITGA4	integrin, alpha 4 (antigen CD49D, alpha 4 subunit of VLA-4 receptor)	7
GNAS	GNAS complex locus	7
TCF7L2	transcription factor 7-like 2 (T-cell specific, HMG-box)	6
MET	met proto-oncogene (hepatocyte growth factor receptor)	6
ITGB7	integrin, beta 7	6
GRM5	glutamate receptor, metabotropic 5	6
GRIN1	glutamate receptor, ionotropic, N-methyl D-aspartate 1	6
ADCY5	adenylate cyclase 5	5
ITGB3	integrin, beta 3 (platelet glycoprotein IIIa, antigen CD61)	5
GRM1	glutamate receptor, metabotropic 1	5
GRIN2A	glutamate receptor, ionotropic, N-methyl D-aspartate 2A	5
GRIN2B	glutamate receptor, ionotropic, N-methyl D-aspartate 2B	5
NTRK1	neurotrophic tyrosine kinase, receptor, type 1	4
BCL2	B-cell CLL/lymphoma 2	4
CTNNA3	catenin (cadherin-associated protein), alpha 3	4
RPS6KA2	ribosomal protein S6 kinase, 90kDa, polypeptide 2	4
RPS6KA3	ribosomal protein S6 kinase, 90kDa, polypeptide 3	4
MAOA	monoamine oxidase A	4
MAOB	monoamine oxidase B	4

# Pathways = number of pathways (in the autism pathway network) in which each gene participates. Table only displays those genes participating in more than 4 pathways.

### Genes with most participation in pathways

To investigate the involvement of each ASD gene in the pathway network, we counted the number of pathways in which each gene participates (Table 3). We found that *MAPK1 (ERK2)*, *MAPK3 (ERK1)*, *HRAS*, *PRKCB* and *BRAF* are the most involved genes, in that they participate in 14–23 out of 40 enriched pathways/collections.

### Coherence between pathway with most interactions and genes with maximal participation

In addition to finding that the MAPK pathway has the highest number of interactions with other pathways in our pathway-pathway interaction map, we also noted that the above-listed genes we separately identified using MSigDB as involved in the most enriched pathways are all according to KEGG Pathway Database specifically involved in the MAPK signaling pathway. We interpreted this coherence across genes, pathways and methodologies as suggesting a very high level of involvement of the MAPK signaling pathway, together with *MAPK/ERK* genes, in the ASD pathway network.

### Integrated MAPK and calcium signaling

**Fig 3:**
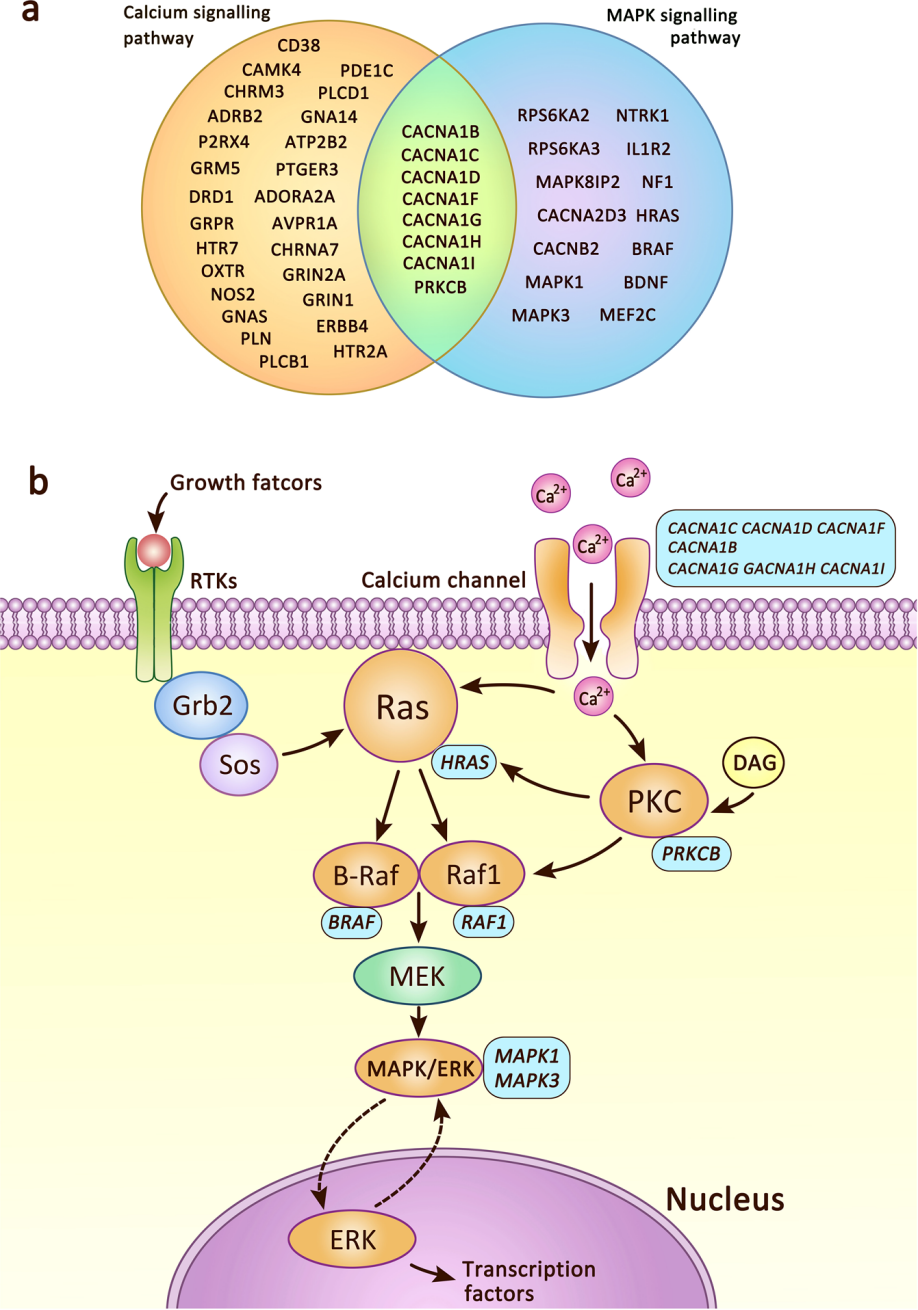
Integrated MAPK and calcium signaling in ASDs. a. Overlapping genes in calcium and MAPK signaling pathways. b. The voltage-gated calcium channel mediates the influx of Ca^2+^, which modulates PKC and Ras. Growth factors bind to the extracellular domain of receptor tyrosine kinases (RTKs), and signal molecules Grb2 (growth factor receptor-bound protein 2) and Sos (son of sevenless) are consequently recruited to the internal docking site, resulting in Ras activation. Ras triggers a phosphorylation cascade including Raf and MEK. This leads to ERK activation and translocation to the nucleus, where ERK then activates transcription factors that mediate gene expression. The ASD genes are listed in light blue frames close to the corresponding gene products that are shown in orange.

We further noted that MAPK and calcium signaling pathways overlapped via 8 ASD genes: *CACNA1H*, *CACNA1G*, *CACNA1I*, *CACNA1D*, *CACNA1B*, *CACNA1C*, *CACNA1F*, and *PRKCB* (Fig 3A).

Looking at the function of these overlapping genes can aid in understanding the potential integrated functions of MAPK and calcium signaling, *CACNA1s* genes encode a group of proteins in the family of voltage-gated calcium channel alpha 1 subunits. The *PRKCB* gene encodes protein kinase C (PKC), beta. PKC is a family of serine- and threonine-specific protein kinases that can be activated by calcium and diacylglycerol (DAG) [8]. Based on the common ASD genes in the MAPK and calcium signaling pathways (*CACNA1s* and *PRKCB)*, we narrowed down the overlapping function of these two pathways in ASDs to voltage-gated calcium channels and calcium activated PKC. Calcium channels in many cell types mediate calcium influx in response to action potentials. An increase in concentration of calcium activates PKC, which regulates Ras and Raf1 proteins by phosphorylation [9]. Once activated, Ras proteins bind to and activate the protein kinase activity of Raf kinases, which phosphorylates and activates MEK (Mitogen-activated protein kinase, MEK1 and MEK2). MEK Phosphorylates and activates MAPK. [10] (Fig 3B). In our ASD pathway network, the top 5 highly involved genes—*MAPK1 (ERK2)*, *MAPK3 (ERK1)*, *HRAS*, *PRKCB* and *BRAF*—encode the proteins MAP kinase 1, MAP kinase 3, small GTPase H-Ras, PKC, and serine/threonine protein kinase B-Raf; together they function in the PKC-Ras-Raf-MAPK/ERK process.

This contextual information allows us to discern that the overlapping ASD genes in the MAPK and calcium signaling pathways, together with most involved ASD pathway network genes, function in the following sequential process whose pertinent highlights include: calcium channel mediates calcium influx, and increased calcium level activates PKC, leading consequently to the Ras-Raf-MEK-MAPK/ERK cascade.

Taken together, integrated analysis for MAPK and calcium signaling pathways came together in the process “calcium-PKC-Ras-Raf-MAPK/ERK”.

## Discussion

In this study we performed a descriptive investigation of the relationships between a large set of ASD-associated genes, the pathways and functions in which these genes participate, and the interactions amongst the pathways. We made every effort to eliminate bias, by using only established and freely available databases and established analytics for utilizing them, and by letting the data generated by these analyses speak for themselves rather than weighting the data on the basis of any *a priori* assumptions or as yet incomplete weighting systems. We present our findings as a kind of “demographics” of ASD-associated genes with the hope of generating hypotheses for further investigation, some of which might not otherwise have emerged from the diverse range of ASD genetic data and the analytic approaches currently in use.

The pathway map we generated shows marked interactions amongst the genes and pathways, and participation of pathways with a broad range of functions and diseases by no means limited to those (such as neurological diseases or development) most commonly considered as centrally relevant to ASD. Although our findings were generated through several separate analyses, we were struck by the convergent findings that emerged from these different approaches. Two pathways stood out most strongly amongst the pathways in which the SFARI ASD-associated genes participated: the MAPK signaling pathway which was the most highly interactive, and the calcium signaling pathway, which was the most highly enriched pathway. Of further interest, based on our pathway-gene interaction analyses, the 8 genes that overlap between the MAPK and calcium signaling pathways are involved in the process “calcium-PKC-Ras-Raf-MAPK/ERK”. What might these findings suggest about possible underlying mechanisms and vulnerabilities in ASDs?

### Diversity of pathway groupings

Amongst the functional pathway groupings, it was interesting to note that there was greater proportional involvement of pathways associated with cell signaling and with metabolism than of pathways associated with nervous system or immune function. Even so, we did find a nervous system pathway, “neuroactive ligand-receptor interaction” as well as a nervous system GO category, “nervous system development”, to be among the most enriched pathways and GO groups, respectively, which is consistent with the predominant emphasis on nervous system development in ASD research and clinical practice. Nevertheless, the diversity of pathway groupings and patterns of proportional involvement suggest that the clinically strongly apparent neuropsychiatric phenotypic features in ASDs may be supplemented and potentially significantly driven by underlying more pervasive processes that are not specific to ASD brain or behavior features–nor for that matter to the increasingly appreciated immune system features of ASDs.

Amongst the disease pathways enriched for ASD genes, we found overlaps with several categories of chronic illness. This observation raises intriguing questions about shared underlying vulnerabilities for these various types of diseases some of which also show at least apparent increases, and may shed some light on the mechanisms underlying that portion of increases in prevalence of ASDs that cannot be explained away by increased awareness, changes in diagnostic criteria, earlier age at diagnosis, diagnostic substitution or other confounds [11,12].

### Diversity and overlaps of Gene Ontology groups

The range of involved GO Gene Sets within each of GO groups is also striking, and again suggests that a broad range of processes may contribute to ASD pathogenesis, though clearly not all at the same time in every single person carrying the ASD diagnostic label. The convergence of the most enriched GO categories in each of the three GO groups with the “neuroactive ligand-receptor interaction” pathway is one of several pointers to potential common underlying mechanisms that our investigation uncovered. Notably, many ASD genes that participate in this pathway encode receptors for neurotransmitters such as serotonin, dopamine, gamma-aminobutyric acid (GABA), glutamate and oxytocin, all of which have documented relationships to ASDs (Table 4) [13]. This suggests that the abnormalities in ASDs related to such neurotransmitters may arise at least in part from dysfunction of their receptor activities. This convergence also suggests that the neuroactive ligand-receptor activities on the plasma membrane, perhaps particularly during nervous system development, might be critical for promoting both the development of ASDs and the ongoing brain dysfunctions associated with the condition.

**Table 4:** ASD genes in the pathway neuroactive ligand-receptor interaction.

Gene Symbol	Gene Description
GRM5	glutamate receptor, metabotropic 5
GRM1	glutamate receptor, metabotropic 1
GRIN1	glutamate receptor, ionotropic, N-methyl D-aspartate 1
GRIN2A	glutamate receptor, ionotropic, N-methyl D-aspartate 2A
DRD1	dopamine receptor D1
HTR2A	5-hydroxytryptamine (serotonin) receptor 2A
ADORA2A	adenosine A2a receptor
AVPR1A	arginine vasopressin receptor 1A
CHRM3	cholinergic receptor, muscarinic 3
ADRB2	adrenergic, beta-2-, receptor, surface
CHRNA7	cholinergic receptor, nicotinic, alpha 7
GRPR	gastrin-releasing peptide receptor
HTR7	5-hydroxytryptamine (serotonin) receptor 7 (adenylate cyclase-coupled)
OXTR	oxytocin receptor
P2RX4	purinergic receptor P2X, ligand-gated ion channel, 4
PTGER3	prostaglandin E receptor 3 (subtype EP3)
GRIN2B	glutamate receptor, ionotropic, N-methyl D-aspartate 2B
DRD2	dopamine receptor D2
GRID2	glutamate receptor, ionotropic, delta 2
LEP	leptin
GRM4	glutamate receptor, metabotropic 4
AGTR2	angiotensin II receptor, type 2
CHRNB3	cholinergic receptor, nicotinic, beta 3
CNR1	cannabinoid receptor 1 (brain)
CNR2	cannabinoid receptor 2 (macrophage)
ADORA3	adenosine A3 receptor
DRD3	dopamine receptor D3
GABRA1	gamma-aminobutyric acid (GABA) A receptor, alpha 1
GABRA3	gamma-aminobutyric acid (GABA) A receptor, alpha 3
GABRA4	gamma-aminobutyric acid (GABA) A receptor, alpha 4
GABRB1	gamma-aminobutyric acid (GABA) A receptor, beta 1
GABRB3	gamma-aminobutyric acid (GABA) A receptor, beta 3
GLRA2	glycine receptor, alpha 2
GRID1	glutamate receptor, ionotropic, delta 1
GRIK2	glutamate receptor, ionotropic, kainate 2
GRM8	glutamate receptor, metabotropic 8
HTR1B	5-hydroxytryptamine (serotonin) receptor 1B
MC4R	melanocortin 4 receptor
OPRM1	opioid receptor, mu 1
GABRQ	gamma-aminobutyric acid (GABA) receptor, theta
THRA	thyroid hormone receptor, alpha

### Pathway-pathway interactions, network hubs and cancer

Although it is intriguing and surprising that the most represented disease pathway / collection in our ASD pathway network is cancer pathways, other methodologies have identified multifunctional genes associated with both cancer and nervous system development or disease [14–16]. The significance of MAPK signaling in our findings may contribute a bit more to understand how ASD and cancer might be associated.

The single most interactive pathway in the ASD Pathway Network, MAPK signaling pathway (also known as Ras-Raf-MEK-ERK pathway), has a diverse range of relationships to functions and diseases. It is involved in a variety of fundamental cellular processes such as proliferation, differentiation, migration, stress response, and apoptosis [10,17]. It is extensively researched in cancer studies, as abnormalities in MAPK signaling play a critical role in the development of cancer [18]. Lately, MAPK signaling has been discussed in ASDs [1] and several recent studies have supported a link between MAPK signaling and ASD traits [19–22]. Also of note is that the important role of MAPK signaling in cancer also coincides with our findings of significantly enriched and interactive cancer pathways—collection cancer, which participates in the ASD pathway network (Fig 2).

It is worth considering what the biological overlap might be between ASD and cancer, specifically given the involvement of MAPK signaling. We would suggest that metabolism might bridge this apparently large gap. Our chain of reasoning here includes the following considerations: 1) MAPK signaling plays an important role in metabolism: it responds to changes in the extracellular or intracellular milieu that often affect the metabolism of the cell; in this regard, altered MAPK signaling has been associated with metabolic syndrome [23]. 2) In our ASD pathway network, there are 8 functional metabolism pathways and 1 metabolic disease pathway, which together constitute 22.5% of the network (Table 1, Fig 1). 3) Moreover, metabolic problems do occur in both cancer and ASDs. It is well known that tumor cells have abnormal glucose metabolism [24]. Regarding ASDs, studies have shown that many people with ASDs are at risk of developing or having metabolic problems as comorbidities [25–27], and maternal metabolic conditions have been recognized as contributing susceptibility to ASDs [28–30]. These documented linkages lend enough plausibility to the idea that ASDs and cancer may be either related at a metabolic level—or at least have marked parallels in contributory mechanisms—including potentially through involvement in both of MAPK signaling regulated metabolic processes, for us to suggest that further investigation to more systematically assess this possible linkage via metabolism would be warranted.

### Calcium signaling dysregulation in ASDs

The enrichment analysis of SFARI genes showed the calcium signaling pathway to be the most enriched, statistically significant pathway. This is consistent with the results from recent ASD studies that used genome-wide association studies (GWAS) data for enrichment analysis [5,31]. GWAS examines genetic variants (typically focusing on SNPs (single-nucleotide polymorphisms)) in different individuals to see if any variant is associated with a trait. In contrast, SFARI Gene integrates different kinds of genetic data that are being generated by research studies. The fact that enrichment analyses using two different kinds of data sources both revealed the calcium signaling pathway as highly significant strongly suggests that it is highly involved in the molecular basis of ASDs.

It was not surprising to see that the calcium signaling pathway emerged from these analyses as important for ASDs. Ever since mutation of the *CACNA1C* gene was found to be associated with Timothy syndrome (a syndromic autism) in 2004 [32], neurogenic calcium dysregulation has been studied in ASD research. Another study, which used GWAS data, also supported the association of calcium channel genes with ASD [33]. The importance of the calcium signaling pathway stands out in our data in four major ways: 1) it is the most enriched pathway; 2) it is the second most interactive pathway in the network; 3) Ca^2+^ (calcium ion) participates in the majority of the enriched pathways/collections, and 4) ASD-associated genes in the calcium signaling pathway are highly involved in the network.

It was unexpected to derive a number of cardiomyopathy pathways from ASD genes (Table 1). After literature searching, we found that people with ASDs indeed appeared to be at increased risk for developing heart disease [26], and reduced cardiac parasympathetic activity was found in children with ASDs [34]. A study that analyzed rare coding variation in ASD cases also found that ASD genes were related to congenital heart disease as well as metabolic disorders [35].

How might the neural and cardiac phenomena mechanistically overlap in ASDs? Common vulnerability to calcium signaling abnormalities are a potential link. Calcium signaling has a wide range of important physiological roles including learning and memory, cellular motility, muscle contraction, etc. [36]. In the heart, calcium is crucial for the regulation of contraction, which is vital to heart functioning [37]. The relationship between calcium and cardiomyopathy has been studied for years [38]. In the nervous system, calcium signaling is important in neuronal synaptic transmission and plasticity [36]. Therefore, if something went wrong with calcium signaling systemically, both cardiac and neural problems might ensue. Although identifying an overlap between neural and cardiac genes ought to make us more attentive to potential comorbidities, the overlap may simply highlight how core molecular functions play roles across diverse systems of the body and brain.

### Integrated MAPK and calcium signaling

The common ASD genes in the MAPK and calcium signaling pathways are *CACNA1s* and *PRKCB*. *CACNA1s* genes encode a group of proteins functioning in voltage-sensitive calcium channels, which mediate the entry of calcium ions into excitable cells (e.g. muscle, glial cells, neurons, etc.), and thereafter initiate a variety of calcium-dependent processes, including muscle contraction, hormone or neurotransmitter release, gene expression, cell motility, cell division, and cell death [39]. The protein encoded by *PRKCB* has been reported to be involved in many different cellular functions, which associate with tumorigenesis [40], insulin resistance [41] and diabetic problems such as hyperglycemia-evoked blood-brain barrier damage [42], as well as diabetic nephropathy [43] and retinopathy [44]. We thus note a theme already observed here, in that in addition to their involvement in ASDs, both *CACNA1s* and *PRKCB* are reported to be involved in both cancer development and metabolism-related issues.

That our integrated findings point to the process “calcium-PKC-Ras-Raf-MAPK/ERK” suggests the possibility that disturbances of this process involving the overlapping genes may contribute to ASDs. Notably, the mutation of *RAF1*, which is also an ASD-associated gene, is additionally associated with dilated cardiomyopathy [45] and hypertrophic cardiomyopathy [46], whose two corresponding pathways were found to be enriched and merged into collection cardiac diseases from the pathway network. PKC and Ras-MAPK/ERK signaling are both known to be associated with cancer [18,47] and involved in regulation of metabolism [23,48]. Taken together, we see that the process “calcium-PKC-Ras-Raf-MAPK/ERK” which our findings suggest may play a key role in ASDs, is also involved in cancer, heart diseases and metabolic problems, some of which are already known to co-occur with or lead to increased familial risk for ASDs [25–27].

### A systematic biological framework

**Fig 4:**
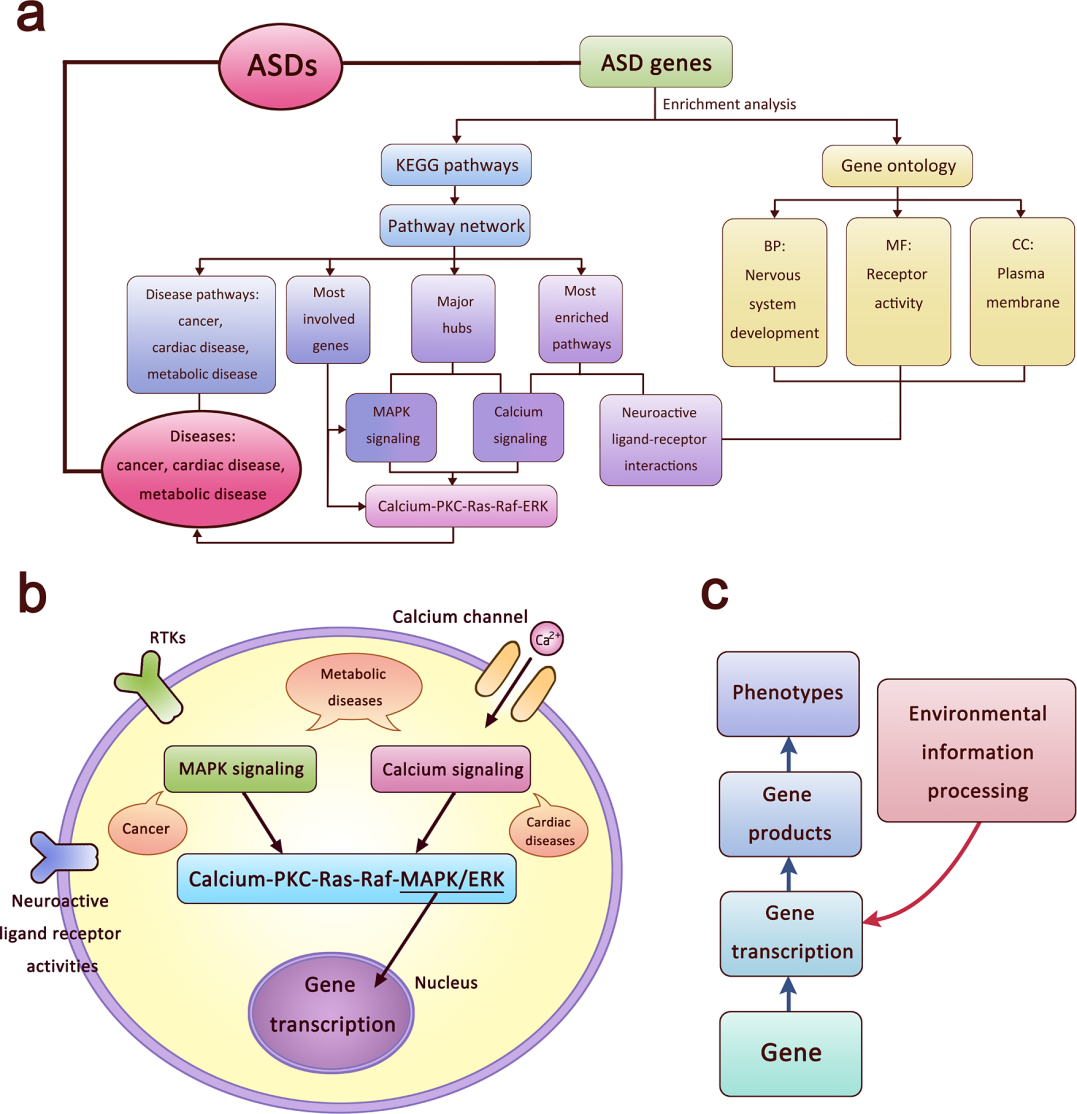
A systematic biological framework. The flow chart in **a,** summarizes the analyses and related results. The conclusions are visualized into a cellular model in **b.** The relationship between genes, gene products, environmental information processing and phenotypes is schematized in **c**.

Based upon the above analyses of pathway-gene relationships, we developed a visualization linking across the different categorical groups using the results as well as the methods from each analysis (Fig 4A and 4B). Based on the GO groups and pathways that we found to be most enriched, we would suggest that it might well be fruitful to allocate serious attention to neuroactive ligand-receptor activities on plasma membrane especially during nervous system development in ASDs. In addition, the MAPK signaling pathway and the calcium signaling pathway, which we found to be most involved in the ASD pathway network, integrate at the process of calcium-PKC-Ras-Raf-MAPK/ERK, and do so via ASD genes that encode proteins functioning in multiple steps. Alterations in the activity of this process can significantly affect its output to multiple systems and may result in serious problems throughout the whole body. Given the increasing appreciation of ASD’s multi-scale, multi-system complexity including its various comorbidities, such a widespread systems impact might be expected. The enriched pathways in cancer, cardiac diseases, and metabolic diseases in the ASD Pathway Network we derived, as well as their interactions with those functional pathways in the ASD pathway network, may also at some level be associated with ASD’s heterogeneous comorbidities. All of this suggests the need for further studies that focus on clarifying how ASDs and other complex diseases are linked, guided by relationships illuminated by interactions among pertinent pathways and reactions.

Based on the whole picture of the conclusions we have derived from our analyses (Fig 4B), we further inferred that “environmental information processing (EIP)” might be important in ASDs. EIP is a category of KEGG pathways (http://www.genome.jp/kegg/pathway.html#environmental, or go to KEGG, click on “KEGG PATHWAY”, and find the category “Environmental Information Processing” listed as number 3 under “Pathway Maps”) to which calcium and MAPK signaling pathways and many of our other enriched pathways belong. EIP means the procedures that cells utilize to react to environmental information and conduct consequent signal transductions. For example, the calcium signaling, MAPK signaling and receptor activities we have been discussing constantly react to signal molecules and changes in the environment outside of cells, with processing of that information leading to a series of reactions including activities inside the nucleus such as gene transcription (Fig 4C). On this basis, environmental factors may trigger intracellular changes and eventually affect gene expression. Environmental risk factors have been discussed in the ASD literature and various studies have suggested their importance [49–51]. Our findings here suggested that EIP might be specifically involved in the underlying mechanism of ASDs as well as gene or gene-function relationship to environmental vulnerability.

### Limitations and potential biases of this study

In spite of our efforts to use unbiased methods, we are aware that bias may remain in our study on account of factors including the limitations of our techniques, the possible biases existing in the current knowledge based databases, and the limited aggregate knowledge within the pertinent fields of research themselves.

Even in a database as carefully constructed as SFARI Gene [52], one can imagine a number of possible sources of bias, such as: 1) The tendency to study genes that are related to genes already implicated in ASDs–for example, the identification of a relationship between CACNA1C and the ASD-related Timothy Syndrome sparked an interest in calcium channels more broadly, leading to an uptick of findings about calcium channel genes in relation to ASDs. 2) The over-representation of longer genes, documented as occurring on the SFARI gene list [53], for reasons that include the higher probability that longer genes will contain a potential mutation, as well as greater technical likelihood of capture for exome sequencing. 3) Underrepresentation in the literature of emerging areas of clear importance. For example, although we now know that more than half of the transcripts in human brain are non-protein coding RNAs (ncRNAs), the field is only beginning to understand the contributions of ncRNA to ASDs and to gene network function, which are therefore presently underrepresented relative to protein-coding in the database. 4) While it is clear that genes in SFARI Gene do not all have the same strength of evidence for inclusion, the Gene Scoring module presently under development for this database that would correct for this [54] is not yet completed, with many genes still unscored, so that there is not yet a systematic, consensual way to correct for this problem.

There are further concerns regarding weighing schemes. While ideally it would be good to evaluate the strength of the association of genes to ASDs before performing the network analysis, it is also possible that such weighting schemes could introduce bias. ASD is hugely heterogeneous, and at present there are no known mechanisms documented to be present in every individual who meets criteria for ASD at the behavioral level. Moreover there are no really strong correlations between any biomarkers and any of the core behavioral features used to define ASD. These are among the reasons why so far as we know, there is no gold standard for ASD gene scoring in the research community, especially when studying the whole massively heterogeneous spectrum. This is particularly important regarding so-called “idiopathic autism” which at present constitutes the majority of individuals with ASDs and whose genetic bases are not yet characterized.

Because of this, we are not able to imagine any criterion for scoring “relevance to ASD” that could be defended as equally relevant to all possible subtypes of ASD. Yet presently, based on DSM-V criteria—which are behaviorally and not biologically or genetically based—so long as someone meets criteria for ASD, they have ASD legitimately—that is, there is no rigorous way to deem some types of fully and reliably diagnosed ASD as being more real than other types. On this basis as well as considerations earlier described, we feel that it would be premature to apply scoring to this set of genes regarding relevance. Moreover, we were concerned that including the score of the ASD genes based on the genetic defects associated with syndromic autism variants could lead to excluding or underweighting potential important genes pertinent to other types of ASD.

There is another potential bias from the KEGG database we used. KEGG PATHWAY is a representation of our knowledge regarding molecular interaction and reaction networks. KEGG’s inclusion of numerous cancer pathways maps in their collections may possibly reflect the intensive research efforts devoted to cancer. To limit the impact of this possible bias upon our outcomes, we applied the redundancy control to the enriched pathways and merged the highly overlapped pathways into collections. However, our finding that the cancer pathway/collection is the most represented disease pathway might still reflect bias.

The outcome of the methods we used to determine the most interactive or hub pathways specifically for ASDs might reflect yet further bias. Although some pathways might interact with more pathways in general and although this might contribute to their levels of interactivity in our network, we did not normalize for this because we could not locate any existing standards to guide such normalization properly and thoroughly. Given the limitations of our knowledge at present, attempted normalization might result in a net addition rather than loss of artifact. As an example, MAPK and calcium signaling pathways play important roles in cells, leading to their high interactivity with other pathways in general, and this may introduce bias in interaction analysis. Even so, it remains striking that MAPK signaling pathways interacted with half (20 out of 40) of the pathways/collections in our ASD pathway network. Whether or not this is a “bias,” it still remains germane to gain further knowledge regarding how these pathways may contribute to ASD mechanisms.

In the end the best protection against bias is to acknowledge its possible sources and to present our data modestly with the aim of contributing to further inquiry.

## Conclusion

In summary, based upon our analyses the MAPK signaling and calcium signaling pathways, particularly the overlapping process “calcium-PKC-Ras-Raf-MAPK/ERK” were found to be strongly associated with ASDs. Both pathways play a central role in a large range of biological processes, whose activities, when abnormal, may compromise biological output and contribute not only to features of ASD themselves but also to a range of other vulnerabilities. It seems quite possible that ASDs may well to a significant extent arise, or emerge, from vulnerabilities related to a set of pleiotropic genes that are also associated with multiple co-morbid systemic issues and that also overlap with other conditions.

The results generated by our emphasis upon gene function leads us to reflect that part of the genetic contributions to ASD pathogenesis may arise from functional disturbances of the processes in which these genes are involved, with such disturbances potentially originating from a variety of reactions and processes changing gene and pathway function, since it is the ensemble of these factors that in the end shapes the phenotype.

Our intention is to give “the lay of the land” within the terms of some of the best resources presently available, and at their current state of development—and not claim discovery of any absolute final truths—we chose to “let the data speak for itself” and offer it up to the community of researchers as one step in a much larger and more collective research enterprise. If our results point any researcher toward a fruitful line of inquiry that might otherwise not have been considered, we will consider that our efforts were well rewarded.

## Supporting Information

S1 Table SFARI genes from Human Gene Module (updated December, 2014). (XLS)

S2 Table Tables and overlap matrix for ASD genes that associated with the enriched KEGG pathways and GO groups. The enrichment analyses data for KEGG and GO (BP, CC and MF) groups were shown in four separate sheets labeled as "KEGG", "BP", "CC", and "MF". Each data sheet contains two parts: the one above is a table of the enriched gene sets, and the one below is the gene set overlap matrix.
(XLS)

S3 Table Pathway-pathway interaction matrix. (XLS)
